# Supplementary material for: The interactome of intact mitochondria by cross-linking mass spectrometry provides evidence for coexisting respiratory supercomplexes
Source: Mol Cell Proteomics. 2017 Dec 8;17(2):216–32. doi: 10.1074/mcp.RA117.000470 (PMC5795388; doi:10.1074/mcp.RA117.000470)
Supplement: Supplemental Data [file supp_RA117.000470_133922_0_supp_23978_fzffwf.zip › spectra_annotation/mito_spectra_annotation/131-1-4-1-7-1.pdf]

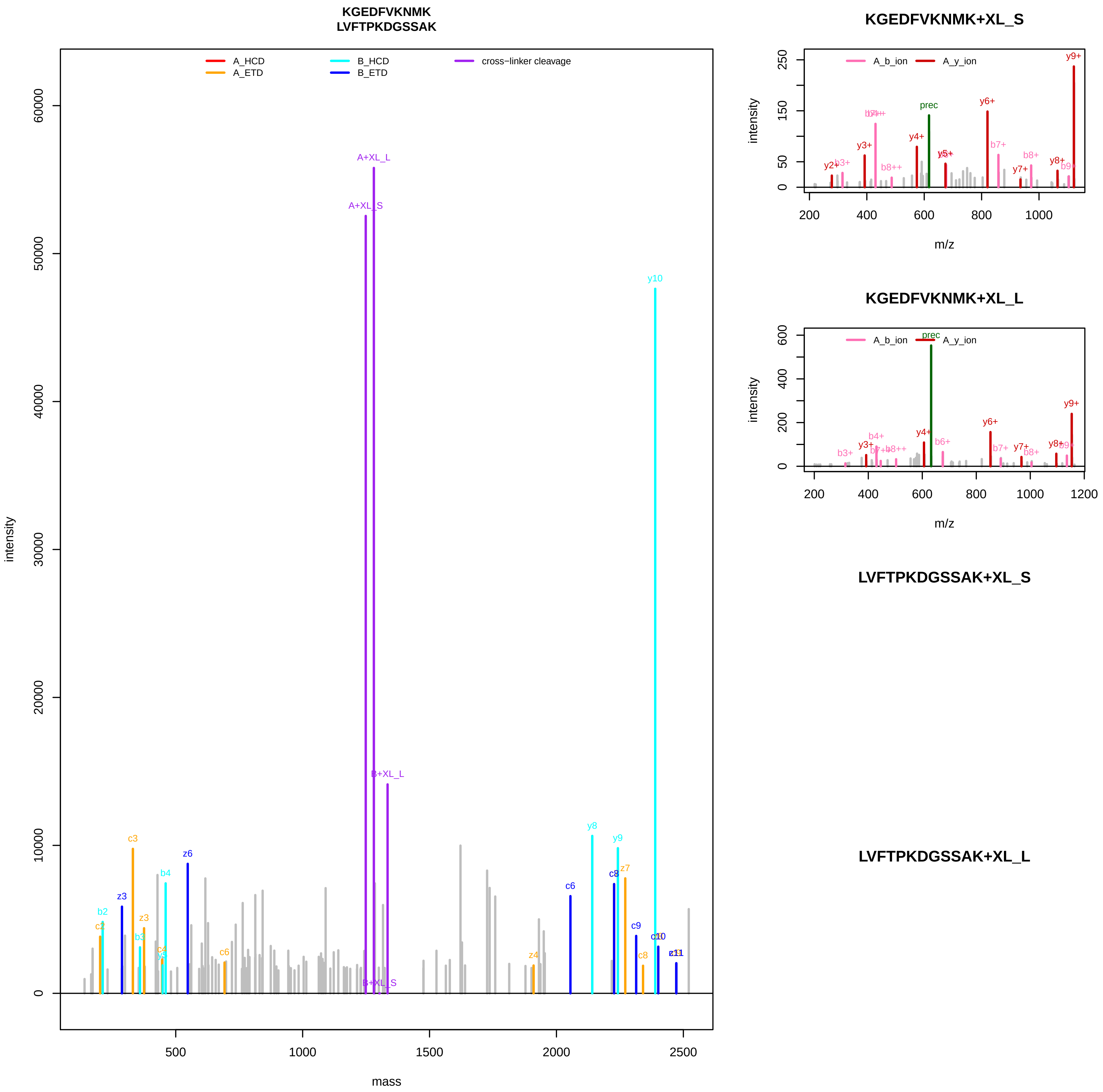
KGEDFVKNMK
LVFTPKDGSSAK
A_HCD
A_ETD
B_HCD
B_ETD
cross−linker cleavage
A+XL_L
A+XL_S
B+XL_L
B+XL_S
y10
y8
y9
c8
z7
c6
c9
c10
c8
z11
c11
z4
c3
z6
b4
z3
z3
b2
c2
b3
c4
y5
c6
intensity
mass
0
10000
20000
30000
40000
50000
60000
500
1000
1500
2000
2500
KGEDFVKNMK+XL_S
A_b_ion
A_y_ion
intensity
m/z
prec
y9+
y6+
y4+
y3+
y2+
b3+
b4++
b7+
b8+
b8++
y5+
y7+
y8+
b9+
0
50
150
250
200
400
600
800
1000
KGEDFVKNMK+XL_L
A_b_ion
A_y_ion
intensity
m/z
prec
y9+
y6+
y4+
y3+
b3+
b4+
b7++
b8++
b6+
b7+
y7+
y8+
b8+
b9+
0
200
400
600
200
400
600
800
1000
1200
LVFTPKDGSSAK+XL_S
LVFTPKDGSSAK+XL_L
